# The Utility of Anti-Covid-19 Desks in Italy, Doubts and Criticism

**DOI:** 10.3390/jfmk6010002

**Published:** 2020-12-30

**Authors:** Marco Bergamin, Giuseppe Musumeci

**Affiliations:** 1 Sport and Exercise Medicine Division, Department of Medicine, University of Padova, Via Giustiniani 2, 35128 Padova, Italy; marco.bergamin@unipd.it; 2 GymHub S.r.l., Spin-off of the University of Padova, Via O. Galante 67/a, 35129 Padova, Italy; 3 Department of Biomedical and Biotechnological Sciences, Anatomy, Histology and Movement Sciences Section, School of Medicine, University of Catania, Via S. Sofia 87, 95123 Catania, Italy; 4 Research Center on Motor Activities (CRAM), University of Catania, 95123 Catania, Italy; 5 Department of Biology, College of Science and Technology, Temple University, Philadelphia, PA 19122, USA

As of the 14th of September, Italy has been considered one of the more susceptible nations in terms of risk of increase for Sars-Cov-2 contagion. In parallel, during the same period, about 5.6-million students physically came back to school from the last lockdown in February [1].

It was concretely hard for the Italian 2020 Covid-19 Emergency Technical–Scientific Committee to set new rules to get students back to class. Arduous decisions were made, especially when some barriers were difficult to be overcome. In particular, the physical distance between students would be simple to be set if classrooms had been permitted to host the same quantity of students with the right “safety space” around them. Before the SARS-CoV-2 lockdown, this safety area was quantified as 1.96 square meters. However, the Italian school system chronically suffers from an infrastructure deficit and this situation is due to the paucity of investments over time [2].

The Italian Ministry of Education (MIUR) decided to invest enormous resources in special desks to promote, in the first instance, higher interpersonal distance in the classrooms to avoid the risk of infection. The stimulation of innovative teaching was a secondary factor in this decision. However, these mobile desks were preferred because they permit a rapid adjustment in the classroom layout. These new desks feature a unique body, with a small inclinable mini-desk on the right side placed over a support frame ending with six wheels (designed to move around the classroom better) and a rubber seat. This desk respects the standards of chairs and tables for educational institutions (EN 1729-1 and EN 1729-2:2012+A1).

This political decision became an opportunity for a bitter media attack on the Italian Government. In fact, the reasons for desk replacement were unjustified since traditional desks were not fixed to the floor and their surfaces were designed to be cleaned by 70% alcohol cleaning agents. From the economic point of analysis, the total provisional expense has not been never disclosed. A single desk costs about 300 euros: the global expected amount, in terms of the quantity of desks replaced, was predicted as being about 2.4 million euros. Furthermore, the cost of old desk disposal has been not computed. This apparently unjustified decision, with high public spending, triggered polemic from the public. Exacerbating this, a further critique was made stressing that these seats were not ergonomically adapted for students. In fact, for example, no adjustments can be carried out by students using these “new-concept” desks. Moreover, taller students with longer lower limbs could involuntarily induce the mobile chair to slip backwards due to the design’s inappropriate leg length. Last but not least, left-handed people will have some trouble writing because of the shape of the table.

Are there any scientific proofs about the unergonomic design of these “anti-Covid-19 desks”?

Are there any scientific proofs showing that these “anti-Covid-19 desks” avoid infection or reduce virus transmission among students?

Obviously no because no studies were yet done on this topic.

Was it really essential to incur this expense to safeguard the health of our children?

This final question probably fits with the Gospel according to Luke “Why do you see the speck in your brother’s eye, but fail to see the beam of wood in your own?”, namely that the Italian government has spent a lot of money to buy new desks while leaving out one of the main problems faced by Italian schoolchildren, which is physical inactivity.

It is well known the incremental decrease in children’s physical activity was observed in Italy despite a decrease in childhood overweight and obesity, the prevalence of which is still among the highest in Europe [3]. This trend is a sort of “syndemic” condition that affects children of all developed countries and that increases drastically during a forced rest period such as the COVID-19 lockdown.

With respect to scientific evidence in the literature showing that the proper use of a backpack and the right preventative measures reduce physical problems and back pain in schoolchildren [4,5], no data has yet been found that suggests or confirms that the new “anti-Covid-19 desks” reduce the risk of contamination.

With this editorial, we hope to stimulate a reflection by experts in the fields of biomechanics, ergonomics and physical activity and our readers. In this optic, we speculated that after a forced rest period of reduced physical activity due to the COVID-19 lock-down, the best public expense to improve the wellbeing of our children should be increasing spending on encouraging safe physical activity instead of buying “anti-Covid-19 desks”. That could lead to a lower risk of children developing chronic conditions related to physical inactivity.

## References

[1] Maugeri G., Castrogiovanni P., Battaglia G., Pippi R., D’Agata V., Palma A., Di Rosa M., Musumeci G. (2020). The impact of physical activity on psychological health during Covid-19 pandemic in Italy. Heliyon.

[2] Steene-Johannessen J., Hansen B.H., Dalene K.E., Kolle E., Northstone K., Møller N., Grøntved A., Wedderkopp N., Kriemler S., Page A. (2020). Variations in accelerometry measured physical activity and sedentary time across Europe@ harmonized analyses of 47,497 children and adolescents. Int. J. Behav. Nutr. Phys..

[3] Lauria L., Spinelli A., Buoncristiano M., Nardone P. (2019). Decline of childhood overweight and obesity in Italy from 2008 to 2016: Results from 5 rounds of the population-based surveillance system. BMC Public Health.

[4] Tobias J.H., Fairbank J., Harding I., Taylor H.J., Clark E.M. (2019). Association between physical activity and scoliosis: A prospective cohort study. Int. J. Epidemiol..

[5] Negrini S., Carabalona R. (2002). Backpacks on! Schoolchildren’s perceptions of load, associations with back pain and factors determining the load. Spine.

